# Anti-Inflammatory and Anticoagulant Activities of Synthesized NSAID Prodrug Esters

**DOI:** 10.1155/2020/9817502

**Published:** 2020-05-23

**Authors:** Murad N. Abualhasan, Motasem Y. Al- Masri, Rufaida Manasara, Lana Yadak, Nael S. Abu-Hasan

**Affiliations:** ^1^Faculty of Medicine and Health Sciences, Department of Pharmacy, An-Najah National University, Nablus, State of Palestine; ^2^Faculty of Science, Department of Biology, An-Najah National University, Nablus, State of Palestine

## Abstract

Paracetamol with ibuprofen or with naproxen are frequently prescribed by doctors in combination. It was found that patients using a combination of NSAID like acetaminophen and ibuprofen experienced less pain. Patients are more compliant if these two drugs are combined in an ester form and given in one dosage form. The esterified prodrugs are hydrolyzed in humans to their active forms. In this study, two esters of paracetamol combined with ibuprofen and naproxen were synthesized as prodrugs. The physiochemical properties of these products were identified. Moreover, the bioactivities of these prodrugs were tested for its anti-inflammatory and anticoagulant activities. The results showed an improved COX inhibition and anticoagulant activity compared with their parent drugs. The synthesized drugs are expected to improve patient's compliance in terms of administration frequency and will have better pharmacokinetic properties with fewer side effects.

## 1. Introduction

Esterification of drugs is very common in drug development; it has many advantages and applications; esterified drugs are used to mask bad taste, it also affects the solubility, bioavailability, and absorption and may increase the patient compliance. Many of the used antibiotics are prodrug esters, and the largest group of these prodrugs was developed to improve oral absorption and bitter tasting masking. Ester linkages of these prodrugs are usually hydrolyzed in human body by esterase enzyme that is present in plasma and various tissues. Esterase enzymes are responsible for the hydrolysis of prodrugs into their active polar water-soluble forms compared with the parent prodrug ester. A prodrug with an ester linkage can also be used to deliver two active drugs; this technique was used in the synthesis of many prodrugs that have anti-inflammatory, analgesic and antipyretic properties [1].

**Figure 1:**
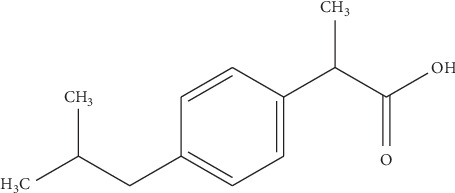
Ibuprofen chemical structure.

Ibuprofen is a nonsteroidal anti-inflammatory drugs (NSAIDs) that is used in the management of mild to moderate pain, fever, and inflammation (Figure 1).

The usual adult dose of ibuprofen is 200 or 400 mg every 4 to 6 hours for the treatment of minor aches, mild to moderate pain, menstrual cramps, and fever. The dose may increase depending on treatment purposes such as in the case of arthritis [2]. It is present in many dosage forms including chewable tablets as well as oral suspension.

**Figure 2:**
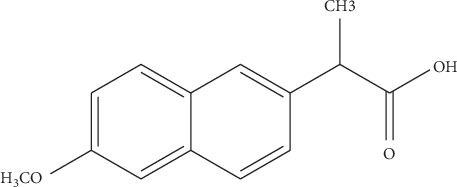
Naproxen chemical structure.

Naproxen is classified as NSAID drug used to relieve arthritis symptoms and may also be used in the treatment of mild to moderate pain, including acute gout and other painful conditions. Side effect of naproxen may include heartburn constipation and nausea [3]. The available pharmaceutical dose of naproxen tablet is 500 mg and 250 mg. The chemical structure is shown in Figure 2.

**Figure 3:**
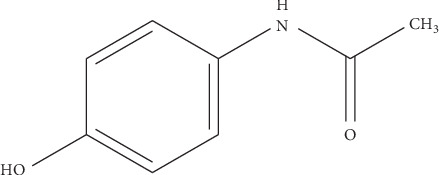
Paracetamol chemical structure.

Paracetamol (Figure 3) is a pain reliever and a fever reducer. It is used to treat many conditions such as headache, muscle aches, arthritis, backache, toothaches, colds, and fevers [4]. The most common available pharmaceutical dose of paracetamol tablet is 500 mg.

There are no reports on pharmacokinetic interaction between acetaminophen and ibuprofen or naproxen, and these drugs are frequently prescribed by doctors in combination. Doctors treating pain in hospital and at home usually consider using paracetamol with other NSAIDs including ibuprofen and naproxan. Moreover, it was found that patients using the combination of NSAID like acetaminophen and ibuprofen experienced less pain during the first 48 h after oral surgery than those using the same daily dosage of either agent alone [5]. Consequently, it will be more convenient to patient if these two drugs are combined in an ester form and given in one dosage form.

In addition to their anti-inflammatory effects, NSAID drugs including ibuprofen were reported to have antimicrobial activity using the minimum inhibitory concentration (MIC) test [6, 7].

Many NSAIDs inactivate cyclooxygenase (COX) and cause platelet dysfunction by inhibiting the formation of thromboxane A2 resulting in antiplatelet effect [8]. Standard coagulation tests include the prothrombin time (PT), and activated partial thromboplastin time (aPTT) assays are general measures of extrinsic and intrinsic clotting pathway integrity, respectively [9, 10].

Esterification is a common chemical reaction in which two reactants, alcohol and acid, form an ester as the final reaction product [11]. Esterification can be achieved by many different synthetic pathways; some of these synthetic routes include Fischer esterification and alcoholysis of acyl chlorides with acid anhydrides. Stegliche esterification is another way of esterification that involves dicyclohexylcarbodimide (DCC) as a coupling reagent using 4-dimethylaminopyridine (DMAP) as a catalyst. The reaction was first described by Wolfgang Steglich in 1978. This reaction generally takes place at room temperature with suitable solvent like dichloromethane [12, 13].

The objectives of this study were to synthesize two prodrug esters that consist of ibuprofen and naproxen separately esterified with paracetamol. The synthesized prodrug esters were identified, and their physicochemical properties were determined using various techniques. Anti-inflammatory and anticoagulant bioactivities of the synthesized prodrugs were also performed.

## 2. Methodology

### 2.1. Reagents

Paracetamol, ibuprofen, and naproxen APIs were given as a gift from Jerusalem Pharmaceutical Company- Palestine. All used reagents were of analytical grade and purchased form liable recourses; these reagents include dichloromethan (DCM), *N,N*′-dicyclohexylcarbodiimide (DCC), *n*-hexan, ethyl acetate, magnesium sulphate (MgSO_4_), 4-dimethylaminopyridine (DMAP), and TLC (DC-Fertigfolien-ALUGRAM®) silica gel (Sigma-ALDRICH/pore size 60A°, 70–320 mesh).

### 2.2. Instrumentation

The instrumentation used in this project includes magnetic stirrer and heater (LM-1003), weighing balance (Adventurer-OHAVS-AR2140), and rotaevaporator (Heidolph-OB2000). IR (infrared) spectra were done using FTIR (Thermo Fisher Nicolet IS5). The UV-VIS spectra were done using the JENWAY-7315 spectrophotometer. Nuclear magnetic resonance (NMR) spectra were obtained using the Bruker Avance (300 MHz) spectrometer, micropipettes (Finnpipette, Finland), incubator (Nuve, Turkey), syringe filter 0.45 *μm* pore size (Microlab, China), microbroth plate (Greiner bio-one, North America), and LABiTec (Germany) for PT and aPTT tests. The Cyclooxygenase (COX) enzyme inhibition test was performed using COX inhibitor screening assay kit No. 560131 Cayman Chemical, USA and was read by Elisaplate Reader (BioRad, Japan).

### 2.3. Chemical Synthesis

The prodrugs of NSAID-paracetamol esters were synthesized by the Steglich esterification procedure using DCC and DMAP. The reaction was performed by reacting 2 mmole of the selected NSAID, which was then dissolved in 20 ml DCM; 1 mmole of DCC (0.412 gm) was added to the solution, and the mixture was allowed to stir for 24 hours. DMAP (40 mg) was then added to the mixture; the mixture was then left to stir for 24 hours. Then, 2 mmole (0.302 gm) of paracetamol was added to the mixture, and the mixture was allowed to stir for 24 hours. The reaction was monitored by TLC using (ethyl acetate : *n*-hexane (70 : 30)) as a mobile phase.

Extraction and chromatographic separation of the synthesized ester was performed by the addition of 50 ml of distilled water to the crude mixture in a reparatory funnel. The organic layer was dried using MgSO_4_. The organic layer was then evaporated under reduced pressure using the rotaevaporator apparatus.

The separation and purification of the drug were achieved by column chromatography. Silica gel was used as a stationary phase, and the drug was eluted using the mobile phase [ethyl acetate : *n*-hexane (70 : 30)]. The extracted residue was dissolved with ethyl acetate and then purified using silica gel column chromatography. Proton NMR tests were performed to confirm product identification. The (NMR) spectra were obtained using the Bruker Avance (300 MHz) spectrometer in CDCl_3_ using tetramethylsilane as internal standard. Chemical shifts were reported in ppm, and coupling constants were recorded in Hz.

### 2.4. Determination of Physicochemical Properties of Synthesized Ester

The melting point of the solid product was determined by using a digital melting point apparatus. The sample was placed in a capillary tube and then inserted to a specific place after the instrument has been calibrated. Two temperature degrees were measured, the first one at the beginning of melting and the other one when almost all of the powder is converted to liquid.

The IR spectroscopy was determined by using an ATR-FTIR. The powder was directly placed in the instrument, and the spectrum of the product was generated. UV-Vis spectrum was measured using the spectrophotometer. Approximately 1 mg of the product was dissolved in methanol, the solution was further diluted ten times; then, the absorbance was measured in the range 200–800 nm.

### 2.5. Bioactivity Evaluation

#### 2.5.1. Anti-Inflammatory Effect by COX Inhibition Activity

COX1 and COX2 inhibition activities were determined using a COX inhibitor screening assay kit No. 560131 by Cayman Chemical, USA. The plate is washed with a buffer solution, and Ellman's reagent, which contains the substrate of acetylcholinesterase, is added to the well. The yellow product of this enzymatic reaction is determined spectrophotometrically in a microplate reader (BioRad, Japan) at 415 nm. The inhibitory assays were performed in the presence of synthesized anti-inflammatory at two concentrations (0.25 and 0.5 *μ*g/ml) and compared with the commercial COX 2 selective anti-inflammatory drug such as celecoxib and for ibuprofen and naproxen, and the results were compared with those in the literature. The anti-inflammatory effect of the tested compounds was evaluated by calculating the percentage inhibition of PGE2 production. The test compound concentration causing 50% inhibition (IC_50_) was calculated from the concentration-inhibition response curve by regression analysis [14].

#### 2.5.2. Anticoagulant Activities (PT and aPTT Tests)

PT and aPTT tests were carried out simultaneously. Blood samples from three healthy volunteers were collected in sodium citrated blood tubes (Vacutainer, BD). Blood samples were then centrifuged for 15 min at 1500 rpm to prepare platelet poor citrated plasma. The LABiTec (Germany) apparatus was used for the determination of PT and aPTT. Equal volumes of drug samples (3000 mg/L) and platelet-depleted plasma were incubated for 5 min at 37°C. The PT test was performed as follows: a 100 *μ*l of tissue thromboplastin (HUMAN) was added to 50 *μ*l of prewarmed mixture, and clotting time was measured. For the aPTT test, 50 *μ*l of rabbit brain extract was added to equal volume of the prewarmed platelet poor plasma-extract mixture incubated for 1 minute after which 50 *μ*l of 0.025 M calcium chloride (HUMAN) was added and clotting time was measured; PBS was used as control [15].

## 3. Results and Discussion

### 3.1. Chemical Synthesis

Esterification reaction using Steglich esterification was successful in the formation of the intended prodrug esters. The number of protons and its chemical shift as shown in the proton NMR prove the success of the synthesis. The proton NMR of the synthesized prodrugs was found as follows:

 
*Naproxen-Paracetamol* (Supplementary Figure 3). ^1^H NMR (300 MHz): *δ* (CDCl_3_): 1.6 (2H, d, *J* = 7.24), 2.09 (3H, s), 3.90 (3H, s), 4.03 (1H, q, *J* = 6.89), 6.88 (2H, d, 8.83), 7.11–7.74 (8H, Ar)
 
*Ibuprofen-Paracetamol* (Supplementary Figure 4). ^1^H NMR (300 MHz): *δ* (CDCl_3_): 0.88 (6H, d, *J* = 6.53), 1.57 (2H, d, *J* = 7.06), 2.06 (3H, s), 1.85 (1H, hep), 2.06 (3H, s), 2.44 (2H, d, *J* = 7.24), 3.90 (1H, q, 3.90), 6.78 (2H, d, *J* = 9.01), 7.11 (2H, d, *J* = 7.95), 7.26 (2H, d, *J* = 7.9), 7.37 (2H, d, *J* = 8.83)

The IR spectrum (infrared) of the product was obtained by the KBr disc technique. IR results of the synthesized and purified compound indicate that this compound is new product that probably has an ester linkage and differs from the starting material.

The IR spectrum of the prodrug product was different from the spectra of parent NSAID. The IR absorption frequencies of the functional group containing a carbonyl (C=O) in an ester has shifted, while the OH group of the paracetamol and the carboxylic acid of the ibuprofen and naproxen has disappeared (Supplementary Figures 1 and 2).

Spectrophotometric measurements were done in the region of ultraviolet and visible light, near-ultraviolet, and near-infrared. In the current study, wavelengths between 200 nm and 800 nm were used. The UV-Vis spectra of the prodrug esters were different from the spectra of the parent NSAIDs. The results of melting points and other physicochemical properties of the synthesized prodrugs esters are shown in Tables 1 and 2.

### 3.2. Bioactivity Results

#### 3.2.1. COX Inhibitory Activity

The enzyme inhibition activity of the synthesized prodrugs was carried out using the ELISA kit. The IC_50_ of the tested compounds was calculated from percentage inhibition of COX1 and COX2 enzymes. The results showed that the calculated IC_50_ of the synthesized paracetamol-ibuprofen combined drug for COX1 enzyme was found to be 0.53 *μ*g/ml. This compound also showed an inhibition activity towards COX2 enzyme, and the calculated IC_50_ was found to be 2.208 ± 0.04 *μ*g/ml. Paracetamol-naproxen-combined drugs showed a COX1 and COX2 inhibition IC_50_ 1.5 ± 0.02 *μ*g/ml and 1.9 ± 0.05 *μ*g/ml, respectively. The results clearly demonstrate that the prodrug paracetamol-ibuprofen was more selective towards COX1 compared with COX 2. In a previous published literature, ibuprofen showed inhibition activity of 1 *μ*g/ml and 46 *μ*g/ml for COX1 and COX2 enzymes, respectively. Naproxen inhibition activities were 2.2 ± 0.9 and 1.3 ± 0.8 *μ*g/ml for COX1 and COX2, respectively. Moreover, as stated in the previous literature, we found paracetamol alone has less IC_50_ inhibition activity for COX1 and COX2 enzymes compared with our synthesized compound. Our synthesized prodrug clearly demonstrates more inhibition activity towards COX1 and COX2 enzyme than the parent individual NSAID drugs, but it is not selective to COX2 as celecoxib (IC_50_ = 47.5 *μ*g/ml). This indicates that our synthesized prodrugs are expected to improve the anti-inflammatory effect compared with its parent compounds. Additionally, it expected that the patients will be more compliant to proper drug administration by taking the combined drug once rather than taking it in two separate dosage forms [15–17].

#### 3.2.2. PT and aPTT Tests

The prothrombin time (PT) and activate partial thromboplastin time (aPTT) tests were performed for both in house synthesized esters and the parent drugs. The newly synthesized ibuprofen-paracetamol ester showed a comparable PT and PTT to the parent drug Ibuprofen as shown in Tables 3 and 4.

The PT of ibuprofen was (21.3 ± 3.3) and for paracetamol was (21.7 ± 4.52), while for the prodrug ibuprofen-paracetamol was (23.8 ± 6.08). The PT of the synthesized naproxen-paracetamol ester was (21.8 ± 2.86). Thus, the PT was almost the same for all tested drugs.

The PTT test results showed that the prodrug ester ibuprofen-paracetamol was 71 ± 0.9, while the aPTT of the synthesized naproxen-paracetamol ester was 69.9 ± 7.7 and they were also comparable to the parent drugs paracetamol and ibuprofen [9, 18, 19].

The above result indicates that the suggested prodrugs have better COX inhibition activity compared with their parent drugs. The ester form of these drugs makes these drugs not acidic and thus will not produce primary acid insult on ulcer which is usually experienced from other commonly used NSAID. The nonselective NSAID has high incidence of peptic ulcer due to their dual peptic ulcer insult, namely, the carboxylic acid present in their structure as well as their COX inhibition effect. Moreover, the low anticoagulant effect results the synthesized compounds will probably have less bleeding side effect. Overall, these drugs as such will have different pharmacokinetic and pharmacodynamic properties before is hydrolysed to the individual NSAID which require more studies involving toxicity and side effects for the tested compounds.

## 4. Conclusion

We successfully synthesized novel combined esters of combined NSAID. The synthesized drug has improved COX inhibitory and comparable anticoagulant activities to that of the parent drugs. The combined NSAID is expected to improve the patient compliance and pharmacokinetics properties.

## Figures and Tables

**Table 1:** Physiochemical properties of synthesized naproxen-paracetamol ester.

Compound structure	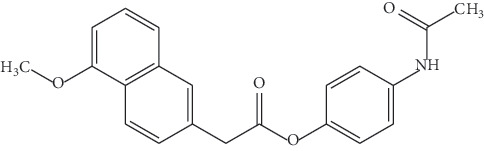
IUPAC name	(S)-4-Acetamidophenyl 2-(1-methoxynaphthalen-6-yl) propanoate
Molecular formula	C_22_H_20_O_4_N
Molecular weight	362
Melting point range	158–160°C
Physical characteristic	Offwhite solid powder
UV-Vis spectra	Maximum is 230 nm

**Table 2:** Physiochemical properties of synthesized ibuprofen-paracetamol ester.

Compound structure	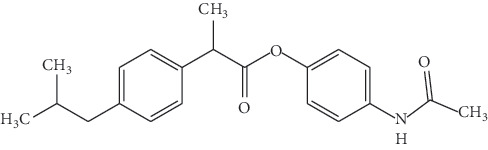
IUPAC name	4-Acetamidophenyl 2-(4-isobutylphenyl) propanoate
Molecular formula	C_12_H_25_NO_3_
Molecular weight	339.43
Melting point range	90–95°C
Physical characteristic	Offwhite solid powder
UV-vis spectra	Maximum is 232 nm

**Table 3:** The PT test results.

Volunteer	PT (seconds)					
	*Normal saline*	*Ibuprofen*	*Ibu-Para ester*	*Paracetamol*	*Naproxen*	*Nap-Para ester*
1	18.5	19.3	19.8	18.5	20	20.4
2	19.4	19.4	20.8	19.4	19.5	19.9
3	19.4	25.1	30.8	27.1	27.3	25.1
Average ± SD	19.1 ± 0.52	21.3 ± 3.3	23.8 ± 6.08	21.7 ± 4.52	22.3 ± 4.36	21.8 ± 2.86

**Table 4:** The aPTT test results.

Volunteer	aPTT (seconds)					
	*Normal saline*	*Ibuprofen*	*Ibu-Para ester*	*Paracetamol*	*Naproxen*	*Nap-Para ester*
1	49	73.7	71.2	73.1	67.4	78.2
2	43	67.6	71.8	62.8	66.2	70.9
3	39.1	59	70	80.2	65.5	60.7
Average ± SD	43.7 ± 4.9	66.8 ± 7.3	71 ± 0.9	72 ± 8.7	66.4 ± 0.9	69.9 ± 8.79

## Data Availability

No data were used to support this study.

## Supplementary Materials

Supplementary Materials This report was created by the ACD/NMR Processor Academic Edition. For more information, visit http://www.acdlabs.com/nmrproc/.
