# Supplementary material for: Anti-Inflammatory and Anticoagulant Activities of Synthesized NSAID Prodrug Esters
Source: Scientifica (Cairo). 2020 May 23;2020:9817502. doi: 10.1155/2020/9817502 (PMC7271289; doi:10.1155/2020/9817502)
Supplement: Supplementary Materials — This report was created by the ACD/NMR Processor Academic Edition. For more information, visit http://www.acdlabs.com/nmrproc/. [file 9817502.f1.pdf]

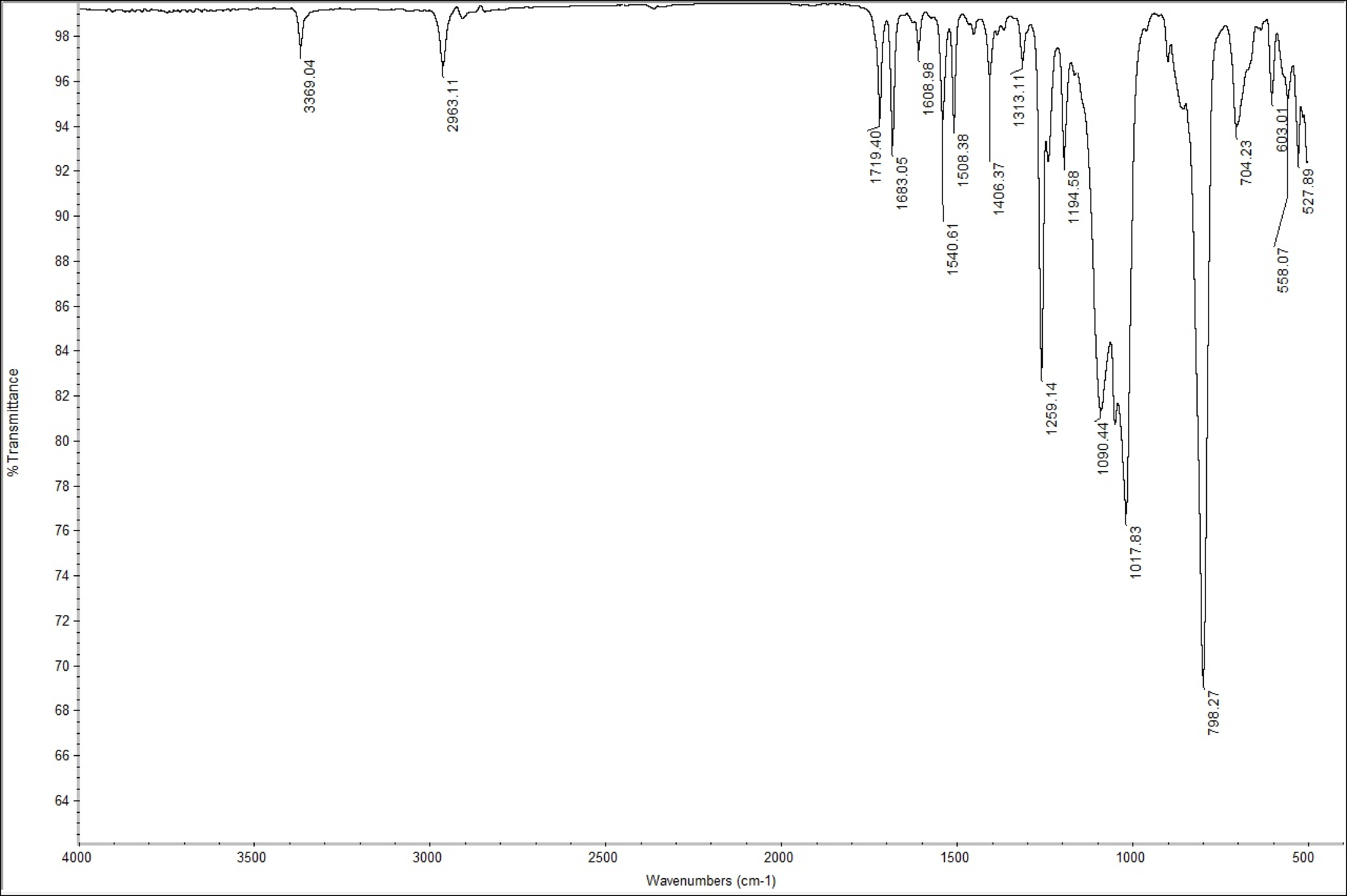

3369.04
2963.11
1719.40
1683.05
1608.98
1540.61
1508.38
1406.37
1313.11
1259.14
1194.58
1090.44
1017.83
798.27
704.23
603.01
558.07
527.89
% Transmittance
Wavenumbers (cm-1)

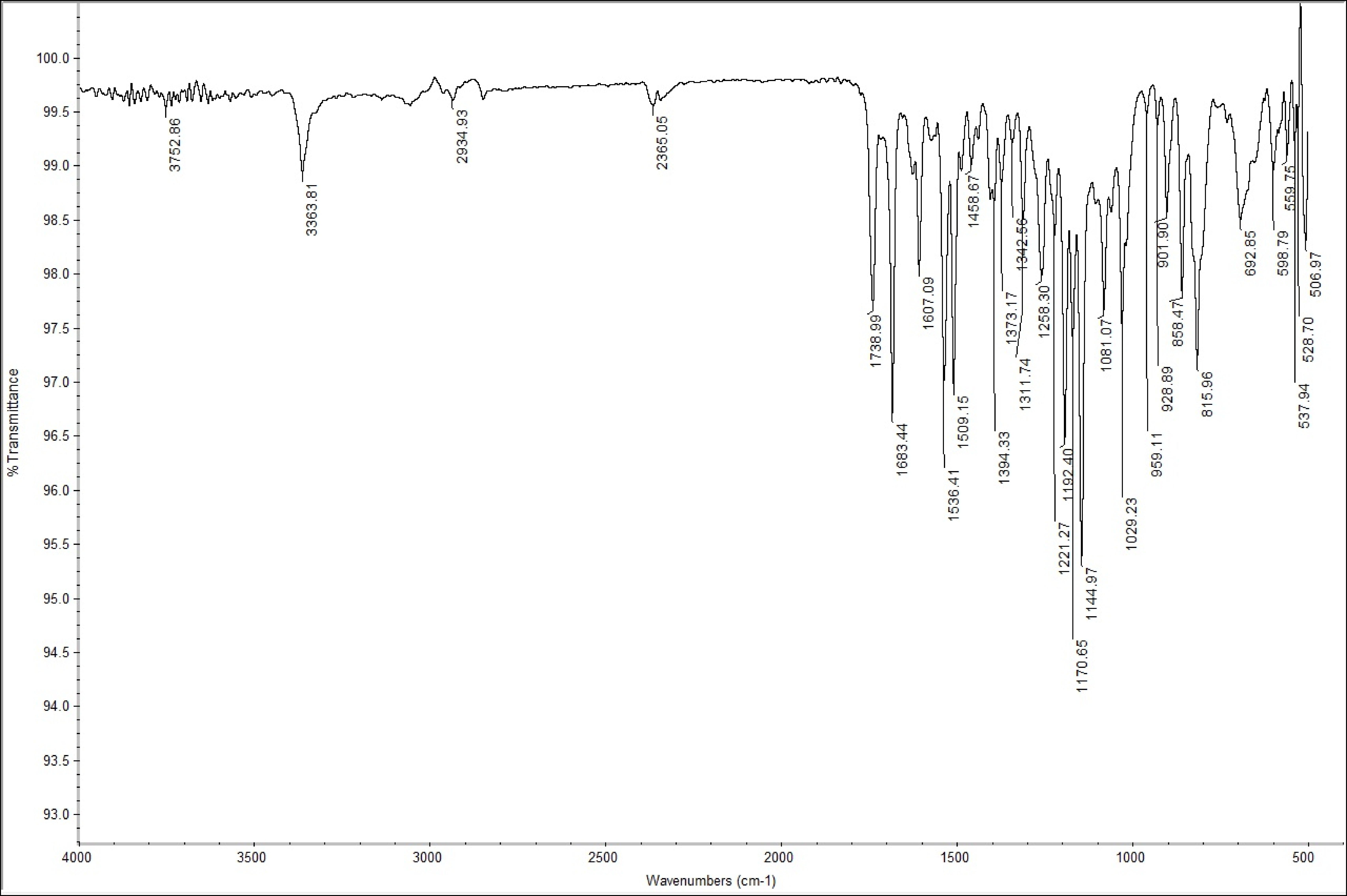

100.0
99.5
99.0
98.5
98.0
97.5
97.0
96.5
96.0
95.5
95.0
94.5
94.0
93.5
93.0
%Transmittance
4000
3500
3000
2500
2000
1500
1000
500
Wavenumbers (cm-1)
3752.86
3363.81
2934.93
2365.05
1738.99
1683.44
1607.09
1536.41
1509.15
1458.67
1394.33
1373.17
1342.56
1311.74
1258.30
1221.27
1192.40
1170.65
1144.97
1081.07
1029.23
959.11
928.89
901.90
858.47
815.96
692.85
598.79
559.75
537.94
528.70
506.97

This report was created by ACD/NMR Processor Academic Edition. For more information go to www.acdlabs.com/nmrproc/

20-Oct-2015 9:13:39 PM

Acquisition Time (sec)	5.6623	***Comment***	PROTON NMR	***Date***	26 May 2015 13:29:04		
Date Stamp	26 May 2015 13:29:04			***File Name***	C:\Users\Abu-Hasan\Desktop\NMR Results\MURAD NMR samples FID\341 Ibu para\fid		
Frequency (MHz)	300.13	***Nucleus***	1H	***Number of Transients***	2	***Origin***	spect
Original Points Count	32768	***Owner***	Administrator	***Points Count***	32768	***Pulse Sequence***	zg
Receiver Gain	228.10	***SW(cyclical) (Hz)***	5787.04	***Solvent***	CHLOROFORM-d		
Spectrum Offset (Hz)	2514.6892	***Spectrum Type***	STANDARD	***Sweep Width (Hz)***	5786.86	***Temperature (degree C)***	25.160

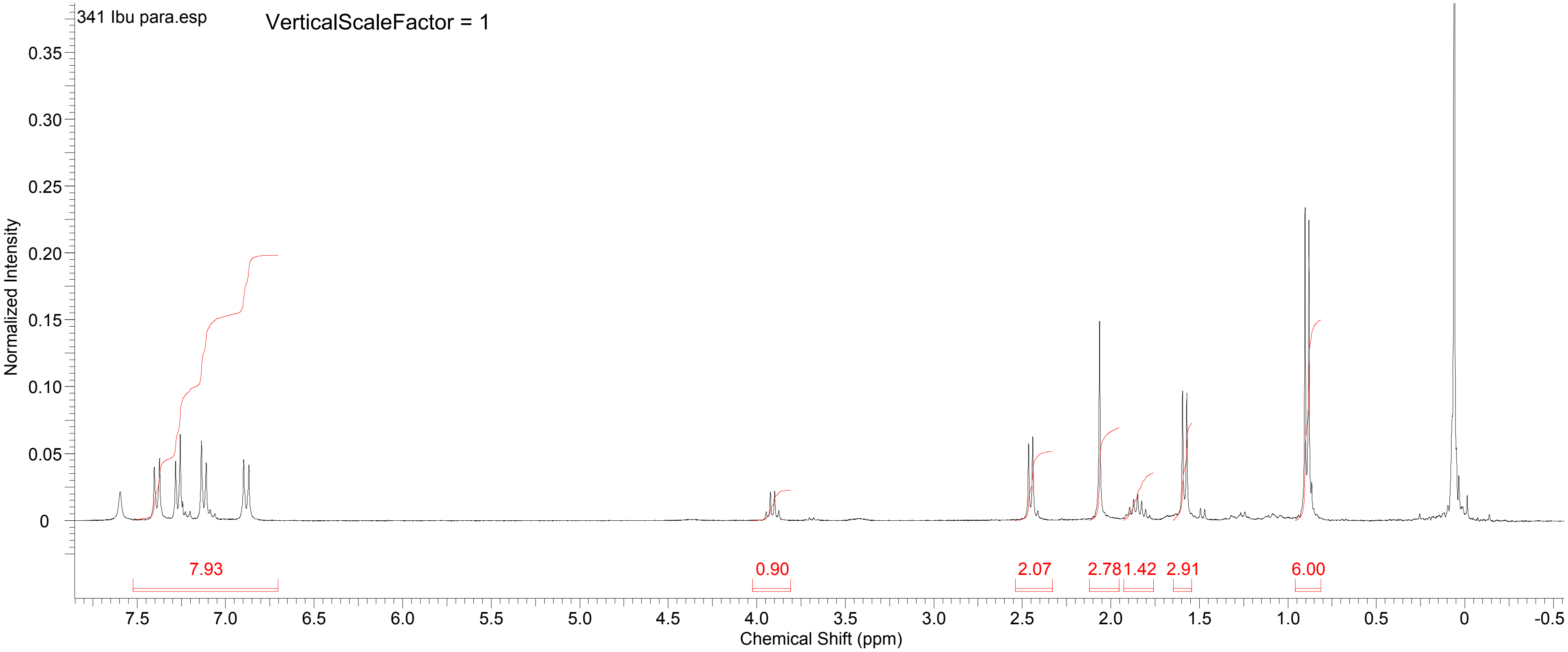

No.	(ppm)	Value	Absolute Value	Non-Negative Value
1	[0.8138 .. 0.95	6.00000000	1.77176848e+8	6.00000000
2	[1.5427 .. 1.64	2.91449881	8.60636160e+7	2.91449881
3	[1.7575 .. 1.92	1.42291367	4.20178920e+7	1.42291367
4	[1.9527 .. 2.12	2.78008389	8.20944160e+7	2.78008389
5	[2.3302 .. 2.53	2.06588745	6.10045680e+7	2.06588745
6	[3.8076 .. 4.02	0.90332675	2.66747640e+7	0.90332675
7	[6.7037 .. 7.52	7.92728662	2.34088608e+8	7.92728662

This report was created by ACD/NMR Processor Academic Edition. For more information go to www.acdlabs.com/nmrproc/

20-Oct-2015 9:04:29 PM

Acquisition Time (sec)	5.6623	***Comment***	PROTON NMR	***Date***	26 May 2015 13:24:48		
Date Stamp	26 May 2015 13:24:48			***File Name***	C:\Users\Abu-Hasan\Desktop\NMR Results\MURAD NMR samples FID\351 Nap Para\fid		
Frequency (MHz)	300.13	***Nucleus***	1H	***Number of Transients***	3	***Origin***	spect
Original Points Count	32768	***Owner***	Administrator	***Points Count***	32768	***Pulse Sequence***	zg
Receiver Gain	812.70	***SW(cyclical) (Hz)***	5787.04	***Solvent***	CHLOROFORM-d		
Spectrum Offset (Hz)	2514.6892	***Spectrum Type***	STANDARD	***Sweep Width (Hz)***	5786.86	***Temperature (degree C)***	25.160

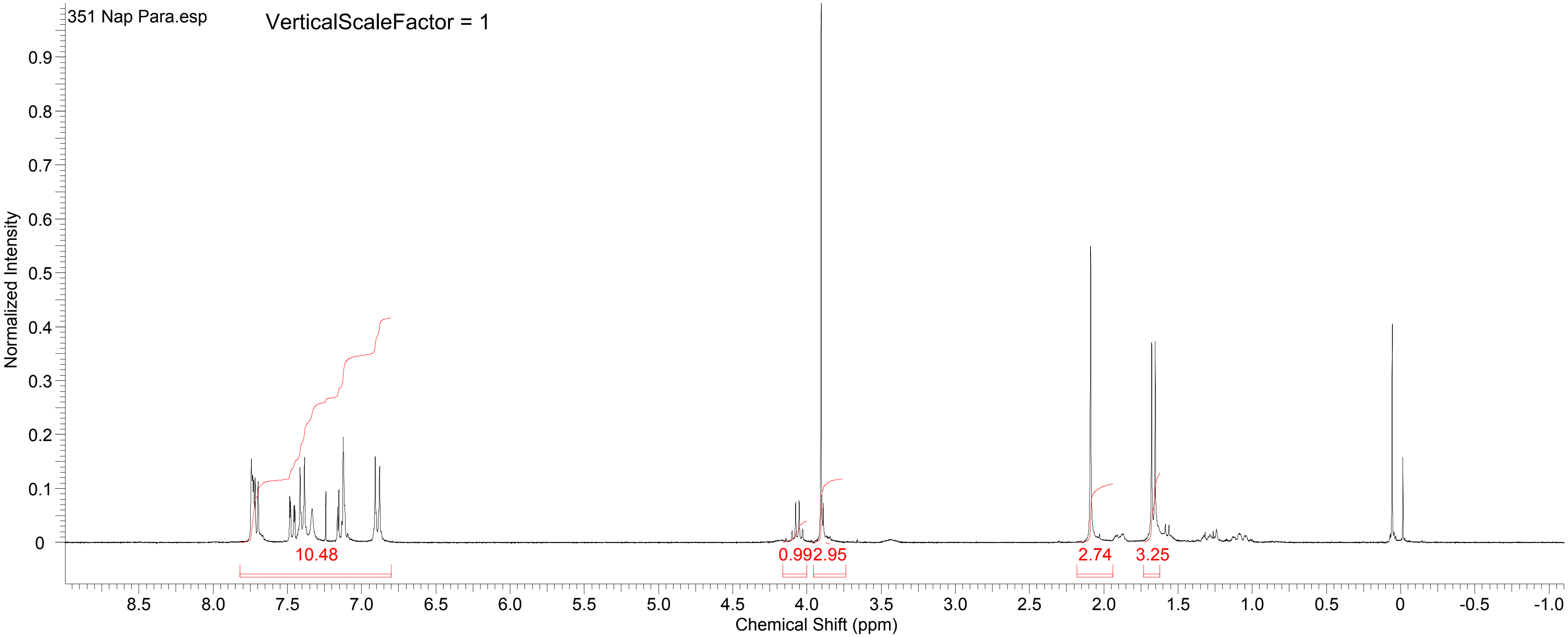

No.	(ppm)	Value	Absolute Value	Non-Negative Value
1	[1.6213 .. 1.73	3.24557924	2.06476336e+8	3.24557924
2	[1.9401 .. 2.18	2.73576188	1.74042928e+8	2.73576188
3	[3.7595 .. 3.97	2.95251870	1.87832496e+8	2.95251870
4	[4.0005 .. 4.16	0.98522657	6.26778640e+7	0.98522657
5	[6.7996 .. 7.81	10.4762134	6.66472768e+8	10.47621346
